# Mutation Screening of the *GLE1* Gene in a Large Chinese Cohort of Amyotrophic Lateral Sclerosis Patients

**DOI:** 10.3389/fnins.2021.595775

**Published:** 2021-05-05

**Authors:** Yanran Li, Bo Sun, Zhanjun Wang, Zhengqing He, Fei Yang, Hongfen Wang, Fang Cui, Zhaohui Chen, Li Ling, Chaodong Wang, Xusheng Huang

**Affiliations:** ^1^Neurological Department of the First Medical Center, Chinese PLA General Hospital, Beijing, China; ^2^Geriatric Neurological Department of the Second Medical Center, National Clinical Research Center for Geriatric Diseases, Chinese PLA General Hospital, Beijing, China; ^3^Department of Neurology, Xuanwu Hospital, Capital Medical University, Beijing, China

**Keywords:** amyotrophic lateral sclerosis, GLE1, loss-of-function mutation, OPTN, oligogenic inheritance of ALS

## Abstract

Amyotrophic lateral sclerosis (ALS) is a fatal progressive neurodegenerative disease involving the upper and lower motor neurons of the spinal cord, brainstem, and cerebral cortex. At least 30 genes have been implicated in familial ALS (fALS) and sporadic ALS (sALS). Kaneb et al. (2015) first carried out a large-scale sequencing study in ALS patients and identified two loss-of-function (LOF) variants in the *GLE1* gene. The LOF mutation-induced disruption of RNA metabolism through the haploinsufficiency mechanism is implicated in ALS pathogenesis. A total of 628 ALS patients and 522 individuals without neurodegenerative disorders were enrolled in this study to explore the *GLE1* gene contribution to ALS in the Chinese population. All 16 exons and the flanking intron of GLE1 were screened by Sanger sequencing. In total, we identified seven rare GLE1 coding variants, including one novel nonsense mutation and six rare missense mutations in 628 ALS patients. The frequency of GLE1 LOF mutations was 0.16% (1/628) among Chinese sALS patients, implying that it is an uncommon genetic determinant of ALS in Chinese patients. Additionally, the rare missense variants in the hCG1-binding domain of GLE1 impairing the distribution of the hGle1B isoform at the nuclear pore complex (NPC) region may be involved in the pathogenesis of ALS.

## Introduction

Amyotrophic lateral sclerosis (ALS) is a fatal progressive neurodegenerative disease involving the upper and lower motor neurons of the spinal cord, brainstem, and cerebral cortex (Robberecht and Philips, 2013; Brown and Al-Chalabi, 2017). Patients with classical ALS die of respiratory failure within 3–5 years of onset after the initial symptoms. A total of 32% of patients with sporadic ALS (sALS) may have a survival time of more than 10 years in the Chinese population (Chen et al., 2015). Additionally, the median survival time of Chinese ALS patients after symptom onset ranging from 59 to 71 months (Chen et al., 2015; Liu et al., 2019) is longer than 44 months among patients in the Western population (Calvo et al., 2017). Approximately 90–95% of the cases present as the sporadic form, and 5–10% of the cases are described as the familial ALS (fALS) form (Byrne et al., 2011). Over 100 genes are related to ALS (Abel et al., 2012)^1^, with more than 30 genes, including SOD1, ALSIN, SETX, SPG11, FUS, VAPB, ANG, TARDBP/TDP-43, FIG4, OPTN, ATXN2, VCP, UBQLN2, SIGMAR1, CHMP2B, PFN1, ERBB4, HNRNPA1, TUBA4A, C9ORF72, CHCHD10, SQSTM1, TBK1, MATR3, NEK1, C21orf2, CCNF, TIA1, and ANXA11, having been replicated in subsequent studies (Mackenzie et al., 2017; Smith et al., 2017; Chia et al., 2018).

Disturbance of the RNA homeostasis is an essential pathological mechanism in ALS. ALS causative genes, including TDP-43, FUS, HNRNPA1, MATR3, and TIA1, with similar protein domains, are RNA-binding proteins (RBPs) (Strong, 2010; Harrison and Shorter, 2017; Zhao et al., 2018). Recently, loss-of-function (LOF) mutations of GLE1 were discovered to be related to ALS (Kaneb et al., 2015). The *GLE1* gene encodes a highly conserved 75-kDa protein with a leucine-rich nuclear export sequence that is essential for the export of mRNAs from the nucleus to the cytoplasm and the regulation of distinct stages of translation (Murphy and Wente, 1996; Kendirgi et al., 2003; Bolger et al., 2008; Alcázar-Román et al., 2010). Kaneb et al. (2015) carried out a large-scale study including 173 fALS and 760 sALS patients using Sanger sequencing and identified two LOF variants in the *GLE1* gene. The LOF variants in GLE1 result in the disruption of RNA metabolism through the haploinsufficiency mechanism, involved in the pathogenesis of ALS. In addition, Nousiainen et al. (2008) previously reported that mutations in GLE1 caused two autosomal recessive disorders, namely, lethal congenital contracture syndrome 1 (LCCS1) (OMIM #253310) and lethal arthrogryposis with anterior horn cell disease (LAAHD) (OMIM #611890). LCCS1 and LAAHD are characterized by early developmental disorder of the spinal motor neuron, suggesting the vital role of Gle1 protein in early motor neuron development. Zhang et al. (2018) conducted a study comprising 230 cases of sALS and 20 cases of fALS. They failed to identify non-synonymous variations in the *GLE1* gene in a study of the Chinese population. Here, a total of 628 patients and 522 individuals without neurodegenerative disorders were enrolled to explore the *GLE1* gene contribution to ALS in the Chinese population.

## Materials and Methods

### Participants

The Chinese patient cohort screened in the study was recruited at the Department of Neurology, Chinese PLA General Hospital (*n* = 368) and Xuanwu Hospital of Capital Medical University (*n* = 260) from December 2010 to October 2019. Patients were examined by two neurologists and diagnosed as possible, probable, and definite ALS based on the Awaji criteria (de Carvalho et al., 2008). The demographic characteristics of the included ALS patients are listed in Table 1. The following clinical information was collected: sex, age of onset, site of onset, disease duration, needle electromyography (EMG), and nerve conduction velocity. The controls (*n* = 522) without neurodegenerative disorders were included. The study was supported by the ethics committee of the Chinese PLA General Hospital and Xuanwu Hospital of Capital Medical University. The participants consented to the publication of clinical information and provided informed consent.

**TABLE 1:** Demographic characteristics of ALS patients.

Variable	
Median age at onset	52.6 ± 10.7 years (17–87)
Sex ratio	1.87:1
Site of onset (percentage)	Bulbar onset: 18.8% (118/628)
	Limb onset: 80.6% (506/628)
	Respiratory muscle onset: 0.5% (3/628)
	Waist onset: 0.1% (1/628)
Diagnosis level (percentage)	Definite level: 50.2% (315/628)
	Probable level: 31.2% (196/628)
	Possible level: 18.6% (117/628)

### Genetic Analysis

Genomic DNA was extracted from the blood samples of all the participants using the TIANamp Genomic DNA Blood Midi kit (TIANGEN, China) according to a standard procedure. All 16 exons of GLE1 (NM_001003722.1) were amplified using polymerase chain reaction (PCR) with the published extension primer sequences listed in Supplementary Table 1 (Kaneb et al., 2015). The purified PCR products were sequenced by an ABI 3730 DNA analyzer (Applied Biosciences, United States). Subsequently, the data were exhibited using the Chromas 1.45 software. The identified hGLE1 variant positions at the genomic level, transcript level, and protein level were based on NC_000009.12, NM_001003722.2, and NP_001003722.1, according to the GRCh37 (hg19) human reference sequence. Identified variations were filtered out with a frequency above 0.05% in the population database mentioned in Table 2. Variants predicted to cause changes in protein function, including nonsense, frameshift, splicing site, insertion, deletion, and missense variants with a minor allele frequency (MAF) < 1%, were selected for genetic and bioinformatics analyses. MutationTaster^2^, CADD^3^, and GERP^4^ were applied *in silico* predictive progress of the identified GLE1 mutations. Patients carrying GLE1 mutations were screened for other ALS-related genes, including SOD1, ALSIN, SETX, SPG11, FUS, VAPB, ANG, TARDBP, FIG4, OPTN, ATXN2, VCP, UBQLN2, SIGMAR1, SQSTM1, CHMP2B, PFN1, ERBB4, HNRNPA1, MATR3, TUBA4A, CHCHD10, NEK1, and CCNF, through the next-generation sequence methods.

**TABLE 2:** Rare coding variants identified in the *GLE1* gene.

Genomic position	cDNA	Protein	Exon	EVS^1^	1000g^2^	gnomAD^3^	dbSNP147^4^	MutationTaster	Polyphen-2	CADD score^5^	GERP++^6^	ALS	Control
**LOF variants**													
131267159	c.75C > A	p.Y25X	Exon 1	0	0	0	–	Disease causing	/	35	3.34	1/628	0/522
**Rare missense variants**													
131267089	c.5C > G	p.P2R	Exon 1	0	0.0004	0.0009588	rs150246404	Disease causing	Benign	27.9	4.31	1/628	0/522
131267142	c.58G > T	p.G20C	Exon 1	0	0	0	–	Disease causing	Damaging	32	5.21	1/628	0/522
131267155	c.71A > G	p.Y24C	Exon 1	0	0	0	–	Disease causing	Damaging	27.7	5.21	1/628	0/522
131271158	c.103G > T	p.V35F	Exon 2	0	0	0.000003977	–	Disease causing	Benign	23.5	3.22	1/628	0/522
131277856	c.370C > T	p.R124W	Exon 3	0	0	0	–	Disease causing	Damaging	34	3.27	0/628	1/522
131285931	c.703C > T	p.R235W	Exon 6	0	0	0.00001953	rs761172264	Disease causing	Damaging	33	5.62	1/628	0/522
131302562	c.1973C > T	p.A658V	Exon 15	0	0	0	–	Disease causing	Damaging	34	5.52	1/628	0/522

*EVS: The Exome Sequencing Project (http://evs.gs.washington.edu/EVS/). 1000g: The 1000 Genome (http://www.ncbi.nlm.nih.gov/Ftp/). dbSNP147: The Single Nucleotide Polymorphism (147) Database (https://www.ncbi.nlm.nih.gov/projects/SNP/). gnomAD: The Genome Aggregation Database (https://gnomad.broadinstitute.org/). ^1, 2, 3^The frequency of the mutation in the EVS database, the 1000 g database, and the gnomAD database, respectively. ^4^Whether the mutation ever recorded in the dbSNP147 database or not. ^5^CADD phred-like score is a phred-like rank score based on whole genome CADD raw scores. The larger the score, the more likely the SNP has damaging effect. ^6^GERP++ score, the larger the score, the more conserved the site.*

### Subcellular Localization of the Gle1 Proteins Encoded by the Rare Missense Variants

To generate the GLE1 rare variant overexpression plasmids, the open reading frame (ORF) of human Gle1 (NM_001003722) was amplified using the PrimeStar HS DNA polymerase kit (Takara, Japan) as the template. The cDNAs encoding Gle1B wild protein, G20C, Y24C, and A658V were directionally cloned into the *Xho*I (5′) and *Kpn*I (3′) sites of pGV230 (CMV-hGle1B/G20C/Y24C/A658V-EGFP-SV40-Neomycin). To generate the pom121 [nuclear pore complex (NPC)-associated integral membrane protein] overexpression plasmid, the ORF of pom121 (NM_001387691) was amplified using the PrimeStar HS DNA polymerase kit (Takara, Japan) as the template. The cDNAs encoding pom121 were directionally cloned into the *Nhe*I (5′) and *Xho*I (3′) sites of pCV296 (CMV-pom121-mCherry-SV40-Neomycin). HeLa cells were cultured in Dulbecco’s Modified Eagle’s Medium (DMEM) (Gibco, United States) with 10% fetal bovine serum (FBS) (Gibco, United States) at 37°C in 5% CO_2_. HeLa cells were co-transfected with plasmids expressing Pom121-mCherry and either hGle1B-WT (Wild-Type)-EGFP, hGle1B-G20C-EGFP, hGle1B-Y24C-EGFP, or hGle1B-A658V-EGFP using Lipofectamine^TM^ 3000 (Invitrogen, United States). Twelve hours after transfection, the location of the EGFP-tagged protein and mCherry-tagged protein was observed on the Zeiss LSM 700 confocal microscope (Zeiss, Germany).

## Results

**FIGURE 1:**
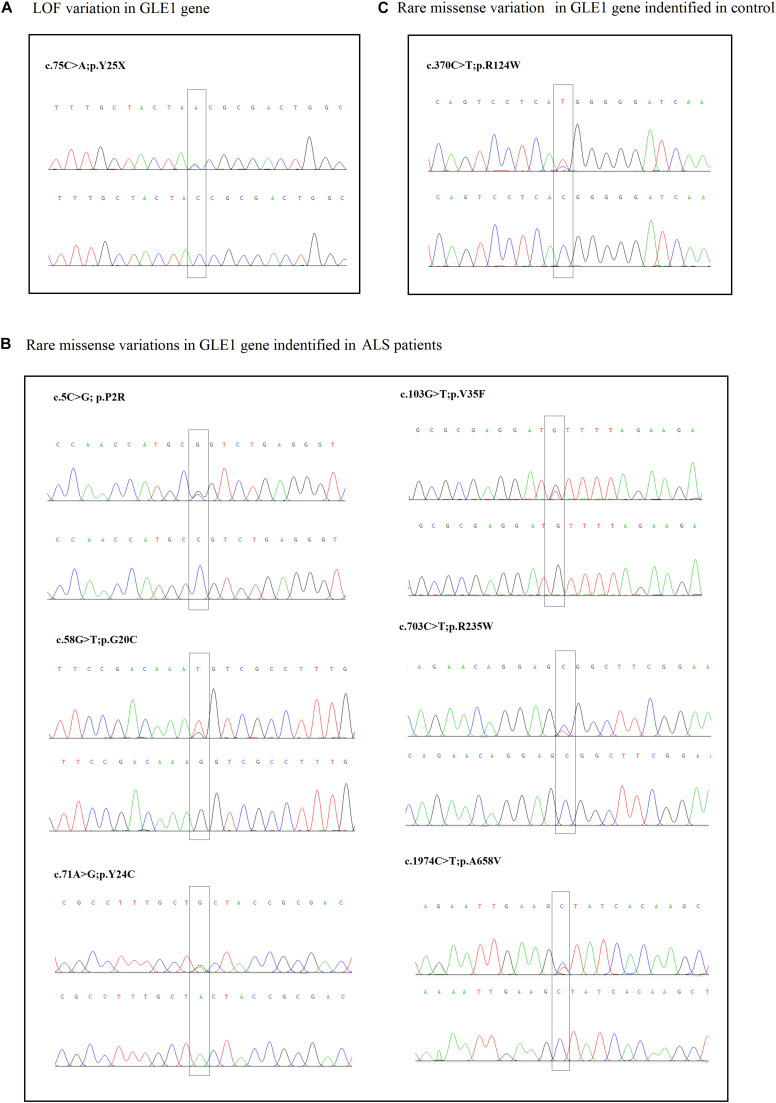
**(A)** The sanger sequencing of the LOF variant identified in sALS patients. **(B)** The sanger sequencing of the rare missense variants identified in sALS patients. **(C)** The sanger sequencing of the rare missense variants identified in control.

**FIGURE 2:**
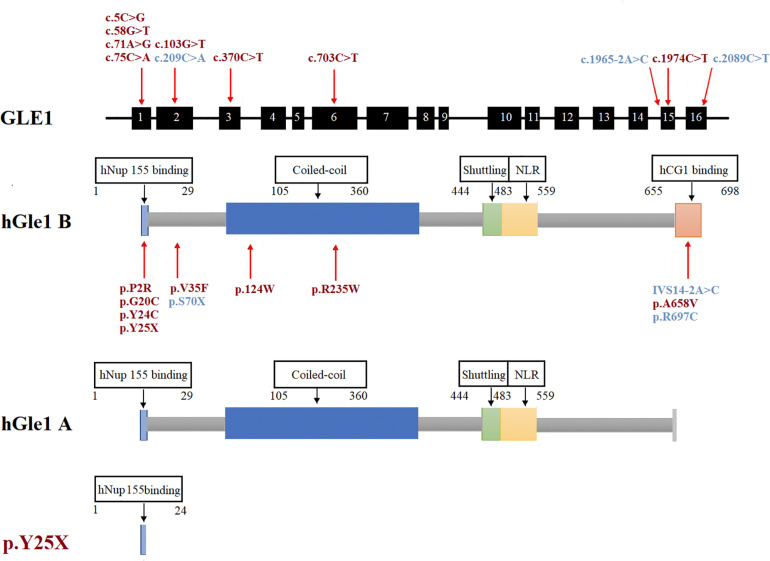
The rare GLE1 variants identified in this study and reported in previous literature. The rare variants identified in this study were marked in red. The rare variants reported in the previous literature on GLE1 were marked in blue.

Seven fALS patients and 621 sporadic patients were included. In total, we identified seven non-synonymous variants (one nonsense mutation and six rare missense mutations) in 628 ALS patients and one missense variant in 522 controls (Figures 1, 2 and Table 2). All the patients with non-synonymous variants are sporadic cases. The clinical characteristics of GLE1 mutation carriers are listed in Table 3.

**TABLE 3:** Clinical characteristics of GLE1 mutation carriers.

Patients	GLE1 mutation	Sex	Sporadic or familial	Site of onset	Age of disease onset (years)	Disease duration (years)	Diagnosis level
347	p.P2R	Female	Sporadic	Spinal	40	10 (dead)	Definite
15R2112	p.G20C	Male	Sporadic	Spinal	37	1 (loss fellow)	Probable
J-42	p.Y24C	Male	Sporadic	Bulbar	57	4.4 (alive)	Definite
15R3994	p.Y25X	Male	Sporadic	Spinal	45	8 (dead)	Definite
17R03845	p.V35F	Male	Sporadic	Spinal	47	2 (loss fellow)	Possible
350	p.R235W	Male	Sporadic	Spinal	51	1.5 (dead)	Definite
J-132	p.A658V	Male	Sporadic	Spinal	48	1.7 (alive)	Definite

The nonsense mutation p.Y25X (c.75C > A), not reported in the ALS and population databases mentioned above, is classified as pathogenic variants based on the American College of Medical Genetics guidelines (ACMG guidelines; Richards et al., 2015). Patients with LOF mutation accounted for 0.16% of ALS cases. The patient carrying the p.Y25X (c.75C > A) mutation exhibited upper limb weakness and muscle atrophy at the age of 45 years, and the symptoms subsequently progressed to the lower limbs and bulbar muscles. The patient died from respiration insufficiency with a disease duration of 8 years. Additionally, the patient carried the p.E273X (c.817G > T) mutation in OPTN, indicating oligogenic inheritance of ALS (van Blitterswijk et al., 2012).

We also detected six rare missense variants in 1.0% (6/628) of ALS cases. The p.P2R, p.G20C, p.Y24C, p.V35F, p.R235W, and p.A658V detected exclusively in the ALS cases and not in the controls fall into the category of variants of uncertain significance according to the ACMG guidelines (Richards et al., 2015; Table 2 and Figure 2). The p.P2R, p.V35F, and p.R235W variants were documented in the population database. The p.G20C, p.Y24C, and p.A658V mutations were not reported in the population or disease databases. Furthermore, the bioinformatic analysis of the missense mutation using the MutationTaster, CADD, and GERP tools showed that the p.G20C, p.Y24C, p.V35F, and p.A658V variants might cause devastating effects on protein function.

**FIGURE 3:**
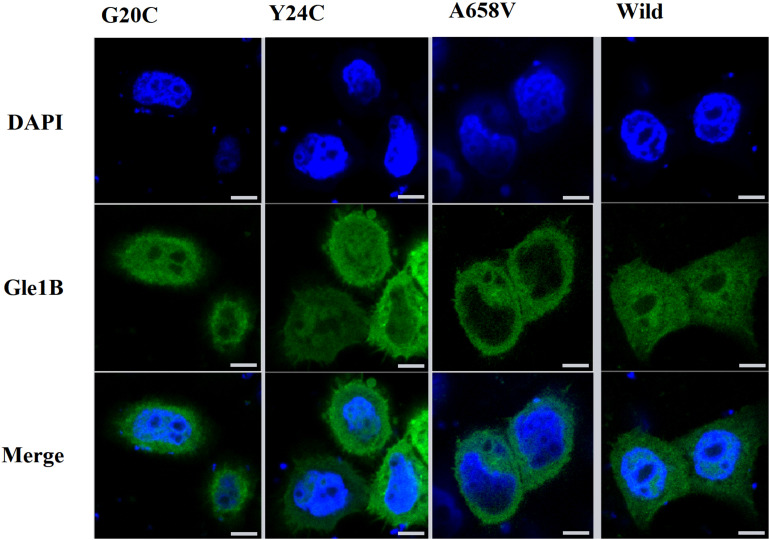
The imaging of HeLa cells transfected with wild-type or mutant EGFP-tagged GLE1 constructs; scale bar: 10 μm.

**FIGURE 4:**
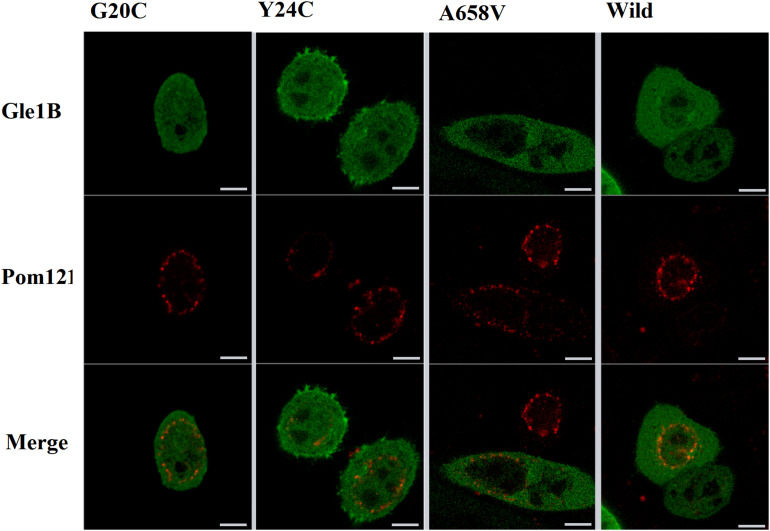
The imaging of HeLa cells transfected with wild-type or mutant EGFP-tagged GLE1 constructs and mCherry-tagged pom121; scale bar: 10 μm.

Therefore, we generated the hGle1B-EGFP and the EGFP-tagged hGle1B variants, including hGle1B-G20C-EGFP, hGle1B-Y24C-EGFP, and hGle1B-A658V-EGFP. When the GFP-tagged hGle1B constructs and the mCherry-tagged pom121 (NPC-associated integral membrane protein) were co-transfected in HeLa cells, we observed that the hGle1B-A658V protein was nearly absent from the nucleus, without apparent co-localization with pom121. Instead, the distribution of hGle1B-G20C and hGle1B-Y24C proteins was similar to the wild-type hGle1B protein, located at the nucleus, cytoplasm, and nuclear envelope with co-localization of pom121 (Figures 3, 4).

## Discussion

A total of eight rare (MAF < 1%) non-synonymous variations in the *GLE1* gene were identified, including seven variations exclusive to ALS patients and one mutation exclusive to controls. We identified the first LOF mutation (c.75C > A; p.Y25X) in the Chinese population, with a frequency of 0.16%. To date, two LOF variants comprising the nonsense mutation (c.209C > A; p.S70X) and splicing site mutations (c.1965-2A > C, hGle1-IVS14-2A > C) have been identified in Caucasian ALS patients. Co-segregation of GLE1 LOF variants has only been confirmed for one splicing site mutation (c.1965-2A > C) in a Finnish pedigree (Kaneb et al., 2015). Zhang et al. (2018) failed to identify non-synonymous variations in the *GLE1* gene in a study including 250 Chinese ALS patients.

The human Gle1 protein (hGle1) plays an indispensable role in the export of mRNAs from the nucleus to the cytoplasm, regulating distinct stages of translation (Murphy and Wente, 1996; Kendirgi et al., 2003; Bolger et al., 2008; Alcázar-Román et al., 2010). There exist two hGle1 isoforms: hGle1A and hGle1B. The hGle1B isoform, localized to the nucleus and cytoplasm and distributed explicitly in the nuclear membrane, especially the NPC region, is 43 amino acids longer at the carboxyl terminus than the hGle1A isoform. In contrast, the hGle1A isoform did not distribute in the nuclear membrane region (Kendirgi et al., 2003). The hGle1B isoform distribution to the NPC region depends on the hNup155-binding domain (1st–29th amino acid) and hCG1-binding domain (655th–698th amino acid) (Kendirgi et al., 2003; Rayala et al., 2004; Figure 2).

We only identified one LOF mutation (c.75C > A; p.Y25X) of the *GLE1* gene in Chinese ALS patients and did not identify LOF mutations among control subjects. Kaneb et al. (2015) reported that the nonsense mutation p.S70X resulted in a deficient expression of the truncated protein through a nonsense-mediated mRNA decay (NMD) mechanism (Kervestin and Jacobson, 2012). In our study, even though the patient’s RNA carrying the p.Y25X mutation was not available to confirm the mechanism of NMD, the premature termination at the very beginning of the Gle1 protein indicated LOF of both the Gle1A and Gle1B isoforms. Another LOF mutation of GLE1, the splicing site mutation (c.1965-2A > C, hGle1-IVS14-2A > C) identified by Kaneb et al. (2015) resulted in the replacement of 44 amino acids at the carboxyl terminus of the hGle1B isoform. This mutation lacking the hCG1-binding structural domain could not bind to NPC and was therefore localized mainly to the cytoplasm but not the nucleus. Furthermore, the disease phenotype was not rescued by expressing the hGle1A and hGle1-IVS14-2A > C proteins alone in a Gle1 knockout zebrafish model, suggesting the indispensable role of the NPC distribution of the hGle1B isoform in the survival of motor neurons. Thus, the identified LOF variants depleted the hGle1B isoform at the NPC region, which is implicated in the pathogenesis of ALS.

We identified six rare missense variants of GLE1, including p.P2R, p.G20C, p.Y24C, p.V35F, p.R235W, and p.A658V, exclusive to ALS patients. The p.G20C, p.Y24C, and p.A658V were not documented in the population or disease databases and were predicted to cause devastating effects on protein function *in silico* analysis. Therefore, we generated the hGle1B-G20C, hGle1B-Y24C, and hGle1B-A658V constructs. The p.G20C and p.Y24C variants, localized to the hNup155-binding domain (Figure 2), did not change the distribution of the hGle1B (Figures 3, 4). In contrast, the hGle1B-A658V protein, encoded by the p.A658V variant in the hCG1-binding domain (Figure 2), was mainly distributed to the cytoplasm but not the nucleus (Figures 3, 4). Furthermore, the p.R697C (c.2089C > T) variant exclusive to ALS cases and the p.I684T (c.2051T > C) variant identified in LCCS1 and LAAHD cases were located at the hCG1-binding domain (Nousiainen et al., 2008; Kaneb et al., 2015). The distribution of hGle1B-I684T protein was similar to the hGle1-IVS14-2A > C protein and the hGle1B-A658V protein, and the steady-state nuclear rim signal intensity for these proteins was reduced (Folkmann et al., 2014; Kaneb et al., 2015). The above hints that the rare missense mutations in the hCG1-binding domain interfered with the distribution of the hGle1B isoform at the NPC region, which is involved in the pathogenesis of ALS.

Notably, the patient with the LOF variant p.Y25X (c.75C > A) in GLE1 also carried another mutation p.E273X (c.817G > T) in OPTN. Maruyama et al. (2010) initially reported that the LOF mutation in OPTN was implicated in ALS patients through the haploinsufficiency mechanism. The p.E273X mutation generated a truncated protein. It failed to bind transcription factor IIIA-interacting protein, myosin VI, and huntingtin, thus interfering with the NF-kappaB neuroinflammatory signaling pathway, Golgi maintenance, and cell autophagy (Chalasani et al., 2009; Toth and Atkin, 2018). Therefore, the nonsense mutation p.E273X in OPTN, not ever reported in the population databases, generating a truncated protein, is also classified as pathogenic variants based on the ACMG guidelines (Richards et al., 2015). This indicates the oligogenic inheritance of ALS, defined as patients carrying more than one gene variant implicated in ALS, including reported pathogenic gene variants and rare functionally predicted deleterious variants (van Blitterswijk et al., 2012; Cady et al., 2015; Morgan et al., 2017). The patient with p.Y25X (c.75C > A) in GLE1 and the patient carrying p.S70X (c.209C > A) reported by Kaneb et al. (2015) were apparent sALS patients without a family history, suggesting the reduced penetrance of GLE1 LOF mutations. It is a pity that the patient’s parents had passed away. We failed to obtain the genomic DNA of the family members to ascertain whether the two mutations mentioned above were *de novo* mutations or not. The two Caucasian ALS patients included a sALS patient carrying the mutation p.S70X (c.209C > A) developed limbs weakness at the age of 61 years and died 2 years later after symptom onset and a fALS proband with the splice site mutation hGle1-IVS14-2A > C (c.1965-2A > C) diagnosed as bulbar onset ALS at the age of 64.5 years and passed away with a disease duration of 1.5 years (Kaneb et al., 2015). The patient carrying p.Y25X (c.75C > A) in the current study exhibits early disease onset but more prolonged survival than the two Caucasian patients described by Kaneb et al. (2015), which is consistent with the epidemiological characteristics of the Chinese ALS and Western population, respectively (Chen et al., 2015; Calvo et al., 2017; Liu et al., 2019).

In conclusion, we identified a total of seven rare GLE1 coding variants, including one novel nonsense mutation and six rare missense mutations in 628 ALS patients. The frequency of GLE1 LOF mutations is 0.16% (1/628) among Chinese ALS patients, implying that it is an uncommon genetic determinant of ALS in Chinese patients. Additionally, the rare missense variants in the hCG1-binding domain of GLE1 impairing the distribution of the hGle1B isoform at the NPC region may be involved in the pathogenesis of ALS.

## Data Availability Statement

The original contributions presented in the study are included in the article/Supplementary Materials, further inquiries can be directed to the corresponding author/s.

## Ethics Statement

The studies involving human participants were reviewed and approved by Ethics Committee of the Chinese PLA General Hospital and Xuanwu Hospital of Capital Medical University.

## Author Contributions

XH and CW devised the study and provided fund support. YL and BS completed the study and the manuscript. ZW, ZH, FY, HW, and FC collected the clinical information and blood samples of patients. ZC and LL performed the electromyography examination for the patients. All authors contributed to the article and approved the submitted version.

## Conflict of Interest

The authors declare that the research was conducted in the absence of any commercial or financial relationships that could be construed as a potential conflict of interest.

## Footnotes

1 http://alsod.iop.kcl.ac.uk

2 http://www.mutationtaster.org

3 http://cadd.gs.washington.edu

4 http://mendel.stanford.edu/sidowlab/downloads/gerp/index.html
