# Supplementary material for: Mutation Screening of the GLE1 Gene in a Large Chinese Cohort of Amyotrophic Lateral Sclerosis Patients
Source: Front Neurosci. 2021 May 5;15:595775. doi: 10.3389/fnins.2021.595775 (PMC8131544; doi:10.3389/fnins.2021.595775)
Supplement: Supplementary file 1 [file Table_1.DOCX]

Supplementary Table 1. The primer sequences

| Exon | Forward primer | Backward primer |
| --- | --- | --- |
| 1 | GTGCAGGCGTTAGGGGC | GAAGTCAAGAAATAAATGCAGGTTG |
| 2 | TAGGCCTTCTTAGATGTTCAGTAA | TATTTCCCCTAGGGTATGGTCACT |
| 3 | ACACCCGGCCTGAGGATAGAGTA | TAGCATACAGTGCCCATTCCTACG |
| 4 | GTGTTAGGGGCCGATGGGCAGGTAGC | AAGCCTCCCTCTTTGTGTTCCTTTGGTTGT |
| 5 | CATAACGAGACCCCATCTCTAAG | TCCCAAGGTTAGTCATACACAGG |
| 6 | ACCTTGGGATGTCCTCTTCC | CAAGTAATGTTTGGTATTTCCCC |
| 7 | AACTATGGATGTGACAGTAAGTTGG | TCACGCAAAGTGTTGGAATC |
| 8 and 9 | TTTTGTGCCTGTTTCCTTTTC | TGTACCCGATCCAACTGTAAAC |
| 10 | TCTGTAGGGCAGCACAGC | CTCCTCGCCTTGTTTCTGAG |
| 11 | AGAGGTATGGGAAGCAGAGG | TCAAGGTACGTAATAGGAGCTGTG |
| 12 | ACAAACAGGCATGCACCAC | TTCCCTAATAATCAACATTCAGTCC |
| 13 | GGTGCTAGGGCTGGATG | AAAATGTATGACACTGGAACCTC |
| 14 | GTTCAGTGGCATTGTGGTTG | ATCAACGAGAGGGACTGGAG |
| 15 | AGTTCAAGGCCTCGGTGAG | AGAAGGGTTTGGAAGAACTAAGG |
| 16 | CAGAACCATCAGTCTTACTGGC | CCCATGAAAATCTCCTCTCC |
